# Gene expression of *Nectin‐4* and its clinical significance in dogs with primary lung adenocarcinoma

**DOI:** 10.1002/vms3.895

**Published:** 2022-07-29

**Authors:** Kei Tamura, Kumiko Ishigaki, Orie Yoshida, Terai Kazuyuki, Naoki Sakurai, Tatsuya Heishima, Tomoko Fujiyuki, Chieko Kai, Kazushi Asano

**Affiliations:** ^1^ Laboratory of Veterinary Surgery, Department of Veterinary Medicine College of Bioresource Sciences, Nihon University Fujisawa Japan; ^2^ Laboratory Animal Research Center The Institute of Medical Science The University of Tokyo Tokyo Japan

**Keywords:** canine primary lung adenocarcinoma, *Nectin‐4*, tumour growth factor

## Abstract

**Background:**

Canine primary lung adenocarcinoma (CPLA) is suspected by radiography or computed tomography; however, since there are no tumour markers, early diagnosis is difficult, and the prognosis is poor due to increased tumour volume. Nectin‐4 has been reported to be expressed in human lung, ovarian, and pancreatic cancers and promotes tumour growth. It has been reported to be a tumour marker and prognostic factor, and oncolytic virotherapy is being investigated using nectin‐4 as a therapeutic target.

**Objectives:**

The purpose of this study was to investigate the expression of *Nectin‐4* in CPLA and its clinical significance in dogs with pulmonary adenocarcinomas.

**Methods:**

The relationships between *Nectin‐4* expression and signalling, tumour volume, tumour weight, and prognosis were analyzed in 34 CPLA patients.

**Results:**

The expression of canine *Nectin‐4* (high *Nectin‐4*) was found in 25 of 34 cases (73%), and *Nectin‐4* expression levels did not show any significant associations with gender, body weight, and tumour stage. However, there was a significant positive correlation between *Nectin‐4* expression and tumour volume (*r* = 0.623, *p* < 0.05) and tumour weight (*r* = 0.735, *p* < 0.05). Regarding prognosis, the median survival time was 427 days in high *Nectin‐4* cases and 420 days in cases with no *Nectin‐4* expression.

**Conclusion:**

Our study demonstrated that *Nectin‐4* is highly expressed in CPLA. In addition, nectin‐4 might be a tumour growth factor in CPLA and thus is a promising biomarker for CPLA. Further investigations on nectin‐4 in CPLA are warranted for its diagnosis and novel targets for oncolytic virotherapy.

## Graphical abstract

Nectin‐4 is a cell adhesion molecule and expressed in several tumor cells. This study demonstrated that gene expression of Nectin‐4 was detected approximately 73% (25 of 34 cases) in canine lung adenocarcinoma and correlated with tumor weight and volume. Therefore, Nectin‐4 might be a tumor growth factor in canine lung adenocarcinoma.

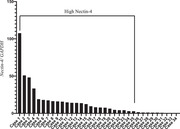

## 1 INTRODUCTION

Canine primary lung tumours are mostly malignant, with adenocarcinoma being the predominant type (Rebhun & Culp, 2013). Clinical symptoms include cough and dyspnoea, but approximately 30% of cases are reported to be asymptomatic (McNeil et al., 1997), and many cases have advanced tumours at the time of diagnosis. In addition, there are no tumour markers, which makes it difficult to detect the tumour at an early stage. Surgical resection is the first choice of treatment, but the prognosis is poor even with surgical resection if metastasis to lymph nodes, other lobes of the lung, or the pleura has occurred (Polton et al., 2008). Furthermore, there are reports that an increase in tumour volume is a factor that worsens the prognosis of canine primary lung adenocarcinoma (CPLA) (Rebhun & Culp, 2013). These findings suggest that the early diagnosis of CPLA may lead to an improved prognosis.

Nectins and nectin‐like molecules (Necls) are calcium‐independent immunoglobulin (Ig)‐like cell adhesion molecules. Nectins comprise four members (1, 2, 3, and 4), and these have been found to be essential contributors to the formation of cell‒cell adhesions (Takai et al., 2008). In addition, they regulate cellular activities such as motility, proliferation, and survival (Takai et al., 2003). Each nectin has a variety of functions and interacts independently or with each other. Nectins‐1, 2, and 3 are expressed in various tissues, and nectin‐4 has been found to be highly expressed in tissues such as the placenta, vagina, and skin but not in normal lung tissue (Fabre et al., 2002; Reymond et al., 2001). On the other hand, high nectin‐4 expression has been found in more than 50% of human breast, pancreatic, and lung cancer patients (Liu et al., 2021). In human pancreatic cancer, the expression of nectin‐4 has also been reported to be associated with prognosis (Nishiwada et al., 2015). As a tumour marker, elevated serum nectin‐4 has been reported to be useful in human lung cancer (Takano et al., 2009). Clinical trials of antibody‒drug conjugate therapy (Challita‐Eid et al., 2016), targeting nectin‐4 and oncolytic virotherapy using the recombinant measles virus, are being investigated (Fujiyuki et al., 2015) as one of the treatment options.

In veterinary medicine, thyroid transcription factor‐1 (Bettini et al., 2009), which differentiates primary lung carcinomas from metastatic tumours, is being investigated as a tumour marker for canine pulmonary tumours, but it has not been applied in clinical practice.

In veterinary medicine, it is known that canine distemper virus uses nectin‐4 as a receptor (Shin et al., 2022), and furthermore, it has been reported that *nectin‐4* expression is involved in tumour growth in prostate cancer (Salda et al., 2019). However, to our knowledge, no studies have examined the expression of *Nectin‐4* in CPLA. We hypothesized that *Nectin‐4* gene expression in CPLA occurs at a high frequency, similar to that in human lung adenocarcinomas. In addition, this may lead to the study of tumour markers targeting nectin‐4 and the development of treatments with oncolytic virotherapy. The purpose of this study was to determine the gene expression of *Nectin‐4* and its clinical significance in dogs with pulmonary adenocarcinomas.

## 2 MATERIALS AND METHODS

### 2.1 Case collection

Canine patients with a pulmonary mass which underwent lung lobectomy and histologically diagnosed as lung adenocarcinoma were included in the study. All patients were investigated for signalment, and blood tests, radiography, and computed tomography (CT) were performed to identify the region of the lung tumour. Staging was based on the classification of canine lung adenocarcinoma (Lee et al., 2020). Consent for the study was obtained from the owners of all the patients.

### 2.2 Surgery

General anaesthesia was pre‐administered by subcutaneous injection of 0.04 mg/kg atropine sulphate (Mitsubishi Tanabe Pharma Co., Osaka, Japan), followed by intravenous injection of 0.1 mg/kg midazolam (Dormicum; Astellas Pharma Inc., Tokyo, Japan) and 0.1 ml/kg fentanyl citrate‒droperidol (Thalamonal; Daiichi‐Sankyo Propharma Co., Ltd., Tokyo, Japan). General anaesthesia was induced using propofol (Mylan; Mylan Seiyaku Ltd., Tokyo, Japan). Endotracheal intubation was subsequently performed. Each dog was mechanically ventilated with 2.0%–2.5% isoflurane (IsoFlo; Zoetis Japan, Tokyo, Japan) and oxygen. For analgesia, intra‐ and post‐operative continuous drip infusions of remifentanil (5–40 μg/kg/h) (Ultiva; Janssen Pharmaceutical K.K., Tokyo, Japan) and pre‐ and post‐operative intramuscular injections of morphine hydrochloride (0.3 mg/kg each dose) (Takeda Pharmaceutical Co. Ltd. Osaka, Japan) were used.

Prior to surgery, CT was performed, and the volume (cm^3^) of the lung mass was measured in terms of length, width, and height. All patients were approached through an intercostal or median sternotomy depending on the region and size of the individual tumours, and the tumours were resected by lung lobectomy. All resected masses were weighed.

### 2.3 Histopathological examination

For histopathological examination, the resected lung masses were sliced and fixed in 10% neutral‐buffered formalin and subsequently embedded in paraffin for histopathological diagnosis. Sections (4 μm thick) were deparaffinized in xylene and rehydrated in ethanol to water. The slides were then stained with haematoxylin and eosin and histopathologically evaluated.

### 2.4 Prognosis

The median survival time (MST) was defined as the time from staging to tumour‐related death or other causes. The follow‐up period was defined as the interval from staging to the last day of follow‐up or death. Additional follow‐up information was obtained through telephonic interviews and e‐mails with the referring veterinarians. Canine patients that were still alive at the end of the study were censored.

### 2.5 Control sampling

Normal lung tissue for real‐time PCR was collected from five dogs (Beagle, individual numbers: 5FW455, 5FW471, 5FW494, 5FW500, and 5FW504) approved by the ethics committee at our institution. These dogs underwent blood tests, and radiography revealed that they were healthy. General anaesthesia was pre‐administered by subcutaneous injection of 0.04 mg/kg atropine sulphate (Mitsubishi Tanabe Pharma Co.), followed by intravenous injection of 0.1 mg/kg midazolam (Dormicum; Astellas Pharma Inc.) and 0.2 mg/kg butorphanol (Butorphanol; Meiji Seika Pharma Inc., Tokyo, Japan). General anaesthesia was induced using propofol (Mylan; Mylan Seiyaku Ltd.). After induction of anaesthesia, a right fourth intercostal incision was made, and the middle lobe of the right lung was resected. A portion of the obtained lung tissue was frozen with liquid nitrogen and stored at −80°C. The animals were then maintained under deep anaesthesia with isoflurane (5%) and euthanized with intravenous potassium chloride (40 mEq/head).

### 2.6 RNA isolation

RNA was isolated from the tumours of canine patients and the lung tissues of five healthy dogs (Beagle, control group) using RNA NucleoSpin columns (Takara Bio Inc., Shiga, Japan) according to the manufacturer's instructions. Tissue samples were homogenized using a motorized tissue grinder (Thermo Fisher Scientific, Waltham, MA, USA). The quality and quantity of RNA were determined spectroscopically using a NanoDrop 1000 spectrophotometer (Invitrogen, Carlsbad, CA, USA).

### 2.7 Reverse transcription and quantitative real‐time PCR

A high‐capacity cDNA reverse transcription kit (Applied Biosystems, Carlsbad, CA, USA) was used for cDNA synthesis according to the manufacturer's instructions.

Using the cDNA generated, the expression levels of *Nectin‐4* as well as that of a housekeeping gene, *GAPDH*, were analyzed by real‐time PCR using TB Green™ Premix EX Taq™ II (Tli RNaseH Plus) (Takara Bio Inc.). The following primers were used: canine *Nectin‐4* (5′‐GTC ACT TCG GAG TTC CAC CT‐3′ and 5′‐TGA GTG TAG CGC CTT CTC TG‐3′.); canine *GAPDH* (5′‐ATG ATT CTA CCC ACG GCA AA‐3′ and 5′‐TCT CCA TGG TGG TGA AGA CC‐3′). Primers were designed using Primer3plus (http://www.bioinformatics.nl/cgibin/primer3plus/primer3plus.cgi) and BLAST (https://blast.ncbi.nlm.nih.gov/Blast.cgi).

### 2.8 Expression analysis of canine *Nectin‐4*

The expression of canine *Nectin‐4* was calculated using the control group for comparison. For comparison, the ΔCt values (target gene Ct value—housekeeping gene Ct value) for each gene were calculated in the control and tumour samples. The ΔCt values of the controls were then averaged, and the ΔΔCt values (tumour sample target gene ΔCt—average control sample gene ΔCt value) were calculated from these values and the ΔCt values of each tumour sample. The relative quantification of canine *Nectin‐4* in the tumour sample, relative to the normal tissue, was determined using the comparative method (2^−ΔΔCt^) (Livak & Schmittgen, 2001). High *Nectin‐4* cases were defined as those with more than double the expression compared to the control group. In contrast, those with less than a twofold increase in expression were defined as having low *Nectin‐4* expression.

### 2.9 Statistical analysis

Correlations between canine *Nectin‐4* expression and clinical parameters were analyzed using the Mann‒Whitney *U* test and Spearman correlation, and Kaplan‒Meier survival curves were plotted and compared using the log‐rank test. All statistical analyses were performed using the GraphPad Prism 8.0 software package (GraphPad software, Inc., San Diego, USA).

## 3 RESULTS

### 3.1 Case characteristics

Thirty‐four dogs were included in this study. Gender was evenly distributed in this cohort of animals: 15 males (12 castrated) and 19 females (18 spayed). The median age and body weight were 11.9 (9–11) years and 8.8 (2.8–38.3) kg, respectively. The breeds were Miniature Dachshund (*n* = 8), Toy poodle (*n* = 4), mixed (*n* = 4), miniature schnauzer (*n* = 3), Chihuahua (*n* = 3), French bulldog (*n* = 2), Welsh Corgi (*n* = 2), Border Collie (*n* = 2), Golden Retriever (*n* = 1), Bernese Mountain Dog (*n* = 1), Norfolk Terrier (*n* = 1), Shih Tzu (*n* = 1), Pekinese (*n* = 1), and American Cocker (*n* = 1). Clinical symptoms included cough in 16 cases (47%), dyspnoea in three cases (8%), anorexia in one case (3%), diarrhoea in one case (3%), and no symptoms in 13 cases (38%). The asymptomatic case was incidentally found to have a pulmonary mass on radiography examination. The most common region of tumour occurrence was the caudal lobe of the left lung (29%), and all patients underwent lung lobectomy with intercostal thoracostomy. The stage classifications were 7, 20, 2, and 5 cases in stages 1, 2, 3, and 4, respectively.

### 3.2 Expression and correlation of *Nectin‐4* in CPLA

**FIGURE 1:**
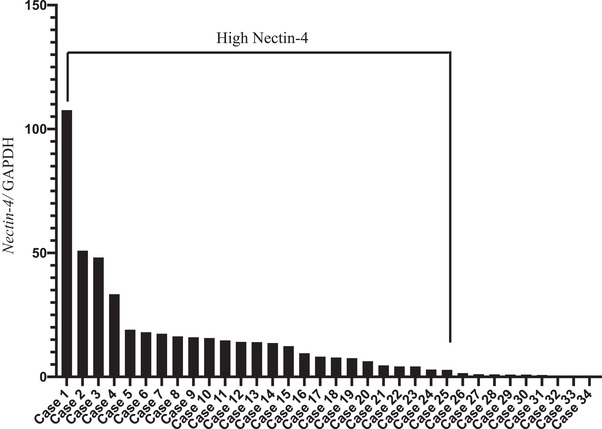
Expression level of canine *Nectin‐4* in canine primary lung adenocarcinoma (CPLA) cases. Real‐time PCR analysis of dog *Nectin‐4* in 34 CPLA cases. Data were normalized to GAPDH and compared to expression levels in normal lung tissue, expressed as 1 (control).

**FIGURE 2:**
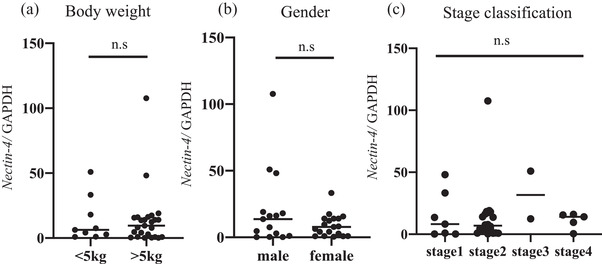
Comparison of canine *Nectin‐4* expression by body weight (a), gender (b), and stage classification (c). The bars indicate the median.

**FIGURE 3:**
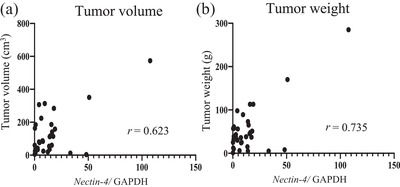
Correlation between canine *Nectin‐4* expression level and (a) tumour volume (cm^3^, *p* < 0.05, *r* = 0.623) and (b) tumour weight (g, *p <* 0.05, *r* = 0.735). Expression of canine *Nectin‐4* was normalized to GAPDH.

High *Nectin‐4* levels were found in 25 of 34 cases (73%) (Figure 1). A comparison of signalment and other parameters in high *Nectin‐4* and low *Nectin‐4* cases is summarized in Table 1. In the cases with the highest expression, there was a marked 107‐fold increase compared to the control group. There were no significant differences in canine *Nectin‐4* expression levels according to body weight, gender, and stage classification (Figure 2). However, the median tumour volume was 113 cm^3^ (3.6–573.8 cm^3^), and the median tumour weight was 53.1 g (1–285 g). There was a significant positive correlation between these parameters and canine *Nectin‐4* expression, with *r* = 0.623 and *r* = 0.735, *p* < 0.05, respectively (Figure 3).

**TABLE 1:** Comparison of signalment data of high Nectin‐4 and low Nectin‐4 groups

	High *Nectin‐4* (*n* = 25)	Low *Nectin‐4* (*n* = 9)
Median age (range)	11.84 (8–19)	12.11 (10–14)
Gender (*n*)	Male (2), casted male (10)	Male (1), casted male (2)
	Female (1), spayed female (12)	Female (0), spayed female (6)
Body weight	8.67 kg (2.8–38.3)	9.14 kg (3.2–15.95)
Breed (*n*)	Miniature Dachshund (7)	Miniature Dachshund (2)
	Mixed (3)	Border Corrie (2)
	Toy poodle (3)	Chihuahua (1)
	Miniature Schnauzer (2)	Mixed (1)
	French bulldog (2)	Miniature schnauzer (1)
	Chihuahua (2)	Toy poodle (1)
	Shih‐ Tzu (1)	American cocker (1)
	Pekinese (1)	
	Bernese Mountain Dog (1)	
	Norfolk Terrier (1)	
	Welsh Corgi (1)	
	Golden Retriever (1)	
Clinical sign (*n*)	Cough (13)	Cough (3)
	Dyspnoea (2)	Dyspnoea (1)
	Anorexia (1)	Diarrheal (1)
	Asymptomatic (9)	Asymptomatic (4)
Stage Classification (*n*)	Stage 1 (4)	Stage 1 (3)
	Stage 2 (15)	Stage 2 (5)
	Stage 3 (2)	Stage 3 (0)
	Stage 4 (4)	Stage 4 (1)
Median tumour volume (range)	131.79 cm^3^ (3.6–573.8)	59.33 cm^3^ (5.6–184)
Median tumour weight (range)	60.56 g (5–285)	32.66 g (1–61)

### 3.3 Prognosis

**FIGURE 4:**
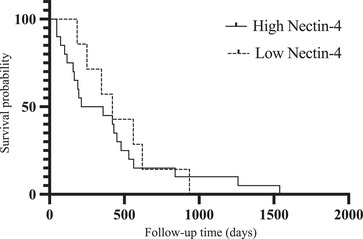
Kaplan‒Meier survival curve of 34 cases with canine primary lung adenocarcinoma (CPLA) based on canine *Nectin‐4* expression status. The solid lines show high *Nectin‐4*, and the dotted line shows low *Nectin‐4*.

The MST in high *Nectin‐4* cases (25 cases) was 427 days and 420 days in low *Nectin‐4* cases (nine cases), with no significant difference between the groups (Figure 4). Regarding the prognosis by stage, the MST for high *Nectin‐4* in stage 1 was 718 days and that for low *Nectin‐4* was 780 days. Stage 2: High *Nectin‐4* was 507 days, and low *Nectin‐4* was 347 days. No significant differences were found in either case. For stages 3 and 4, MST in the expression of *nectin‐4* could not be measured in as few as two and five cases, respectively. The MST for all patients in this study was 749 days for stage 1, 454 days for stage 2, and 131 days for stage 4. MST could not be measured for stage 3 because only two patients were in stage 3. Significant differences were observed in stages 1, 2, and 4 (*p* < 0.05). Survival was confirmed in seven of 34 cases, including one case with the highest expression of *Nectin‐4*.

## 4 DISCUSSION

In this study, canine pulmonary carcinomas are likely to express high levels of *nectin‐4*, as reported in human patients. Furthermore, the expression frequency of *Nectin‐4* in CPLA was as high as 73%. There was no significant difference between canine *Nectin‐4* expression according to gender, body weight, stage classification, or prognosis, but there was a significant positive correlation between canine *Nectin‐4* expression level and tumour volume (cm^3^) and tumour weight (g).

Regarding nectin‐4 and the promotion of tumour growth, a mechanism that activates cell motility has been reported. It has been shown that lamellipodia elongation is essential for cell motility and invasion, which requires the activation of the GTPase Rac1. Rac1 is activated by nectin‐4 (Takai et al., 2001), and in human lung adenocarcinoma, the expression of nectin‐4 activates Rac1 and promotes tumour cell invasion and growth (Takano et al., 2009). It is necessary to analyze whether the expression of *Nectin‐4* in canine CPLA is involved in tumour growth, as in human *Nectin‐4*‐expressing tumours.

The genes for epidermal growth factor receptor and anaplastic lymphoma kinase, which have been found to be mutated in human cancers, have also been studied in canine lung adenocarcinoma (Mariotti et al., 2014), and more recently, human epidermal growth factor receptor 2 protein overexpression in immunohistochemistry has also been found in canine lung cancer (Yoshimoto et al., 2020). Thus, although the characteristics of the cancer are becoming clearer, the diagnosis of CPLA can only be done through radiography or CT scan, with tumour markers not currently clinically applied. On the other hand, cytokeratin 19‐fragment (CYFRA21‐1) and carcinoembryonic antigen have been used as tumour markers in human lung adenocarcinoma (Dong et al., 2016; Zhao et al., 2014), and reports indicate that sensitivity increases when these markers are measured simultaneously with serum nectin‐4 (Takano et al., 2009). In our study, 13 cases (38%) were asymptomatic, and three of the five cases in stage 4 were asymptomatic. Based on these results, it is necessary to increase the use of diagnostic tools such as tumour markers for early diagnosis, as asymptomatic CPLA patients may have stage progression and a worse prognosis. In our study, the high frequency of canine *Nectin‐4* expression suggested the possibility that nectin‐4 may be a useful tumour marker. To our knowledge, there is no ELISA kit that can measure serum nectin‐4 levels in dogs; therefore, further research and development are needed.

Nectin‐4 has also been found to be a receptor for measles virus (MV) infection (Mühlebach et al., 2011), and MV has been reported to infect tumours expressing nectin‐4, inducing tumour regression (Grote et al., 2001). Sugiyama et al. developed a recombinant MV‐SLAMblind (rMV‐SLAMblind) from a wild‐type HL strain. This virus is blind to signalling lymphocytic activate molecule (SLAM), a receptor important for virulence expression (Sugiyama et al., 2013). The rMV‐SLAMblind virus has lost pathogenicity and has been found to have oncolytic effects in human lung adenocarcinoma cells expressing nectin‐4 (Fujiyuki et al., 2015). Lung lobectomy is the treatment of choice in CPLA, but the prognosis is still poor even after surgical intervention when there are metastases to other lung lobes or pleura (McNeil et al., 1997). Furthermore, chemotherapy and molecular‐targeted drugs had little effect. Therefore, it is important to study novel therapies for metastatic cases. Our study may also serve as the basis for the novel treatment of CPLA with rMV‐SLAMblind, as *Nectin‐4* is highly expressed in spontaneous CPLA.

In this study, there was no significant difference between prognosis and canine *Nectin‐4* expression, even when the cutoff for *Nectin‐4* expression was more than twofold. Furthermore, the MST in this study by stage classification showed no difference compared to previous reports (Lee et al., 2020). In addition, there were cases with high *Nectin‐4* expression and large tumour volumes that survived for a long time after surgical resection. Human lung adenocarcinoma is different from the results of our study because nectin‐4 expression has been reported to be a poor prognostic factor (Liu et al., 2021; Takano et al., 2009). However, there is veterinary literature indicating that an increase in tumour volume is a factor that worsens the prognosis of CPLA (Rebhun & Culp, 2013), so further analysis of nectin‐4 expression levels and prognosis is necessary.

A limitation of this study was that the expression of canine *Nectin‐4* was examined genetically, but immunohistochemistry was not performed. Furthermore, the association between histological subtypes of lung adenocarcinoma and *Nectin‐4* expression levels is important but has not been investigated and needs further investigation. The case with the largest tumour volume and weight was significantly different from the other cases, which had a small effect on the correlation factor. Furthermore, since the number of cases is not large, it is necessary to accumulate more cases and investigate the correlation between disease parameters and canine *Nectin‐4* expression in the future.

In conclusion, our study demonstrated that *Nectin‐4* is highly expressed in CPLA and thus is a promising biomarker for CPLA. Further investigations on nectin‐4 in CPLA are warranted for its diagnostic utility and as a potential novel target for oncolytic virotherapy.

## CONFLICT OF INTEREST

The authors declare no conflict of interest.

## ETHICS STATEMENT

All prodecures and experimental protocols were approved by our Institutional Ethical Committee.

## AUTHOR CONTRIBUTIONS

*Conceptualization, data curation, investigation, methodology, visualization, writing—original draft, and writing—review and editing*: Kei Tamura. *Conceptualization, data curation, investigation, methodology, project administration, supervision, writing—original draft, and writing—review and editing*: Kumiko Ishigaki. *Conceptualization, data curation, supervision, writing—original draft, and writing—review and editing*: Orie Yoshida. *Data curation, investigation, methodology, visualization, writing—original draft, and writing—review and editing*: Naoki Sakurai. *Data curation, investigation, methodology, visualization, writing—original draft, and writing—review and editing*: Tatsuya Heishima. *Conceptualization, data curation, investigation, methodology, project administration, supervision, visualization, writing—original draft, and writing—review and editing*: Tomoko Fujiyuki. *Conceptualization, data curation, investigation, methodology, project administration, supervision, visualization, writing—original draft, and writing—review and editing*: Chieko Kai. *Conceptualization, data curation, investigation, methodology, project administration, supervision, visualization, writing—original draft, and writing—review and editing*: Kazushi Asano.

### PEER REVIEW

The peer review history for this article is available at https://publons.com/publon/10.1002/vms3.895.

## Data Availability

The data that support the findings of this study are openly available in Nihon university Repository at http://repository.nihon‐u.ac.jp/xmlui/handle/11263/7.
